# The effects of an acute exercise on executive function in Chinese undergraduate students

**DOI:** 10.3389/fpsyg.2026.1853294

**Published:** 2026-05-19

**Authors:** Zhanshuang Bai, Yuanqi Huang, Linjun Li, Jinhao Ding, Jiamiao Li, Hongxin Li, Liming Zhang

**Affiliations:** 1 School of Tourism and Sports Health, Hezhou University, Hezhou, China; 2 School of Sports Science and Technology, Bangkokthonburi University, Bangkok, Thailand; 3 School of Physical Education and Sport Sciences, Fujian Normal University, Fuzhou, China

**Keywords:** executive function, exercise, HIIT, MIAE, undergraduate students

## Abstract

**Introduction:**

Previous studies have shown that acute exercise may be associated with short-term changes in executive function. However, executive function is a multidimensional construct, and it remains unclear whether acute exercise influences specific components, including inhibitory control, working memory, and cognitive flexibility. Therefore, the present study aimed to examine the effects of acute exercise on executive function in undergraduate students.

**Methods:**

Forty-four undergraduate students were recruited and randomly assigned to a high-intensity interval training (HIIT) group, a moderate-intensity exercise (MIAE) group, or a Control group. Participants in the exercise groups completed an acute exercise, whereas those in the Control group remained in a non-exercise resting condition. Executive function was assessed using cognitive tasks targeting inhibitory control, working memory, and cognitive flexibility. All participants completed the cognitive tasks before and after the intervention.

**Results:**

Analysis revealed no significant Time × Group interaction effects for either inhibitory control or working memory. For cognitive flexibility, the Time × Group interaction was significant, and both exercise groups exhibited enhanced performance, whereas the Control group did not show similar improvements.

**Discussion:**

These findings are generally consistent with previous evidence suggesting that acute exercise may be associated with changes in executive function. The observed patterns in cognitive flexibility may indicate a potential selective association with certain components of executive function. Although some positive trends were observed in specific aspects of executive function among undergraduate students, these changes were not consistently specific to the exercise conditions. In particular, significant Group × Time interactions were observed only for cognitive flexibility, while no significant interaction effects were found for inhibitory control or working memory. Therefore, descriptive results should be interpreted cautiously.

## Introduction

Executive function refers to a set of effortful, top-down control processes involved in the regulation of attention and information processing, including the selection of task-relevant information, inhibition of dominant but inappropriate responses, flexible adjustment of behavior to changing task demands, and the intentional control of thoughts and actions (Diamond, 2013). Executive functions comprise three core subcomponents: inhibitory control, working memory, and cognitive shifting. Although these executive function components are moderately correlated, they are nonetheless dissociable and can operate relatively independently during the performance of complex tasks (Diamond, 2013; Miyake et al., 2000). Many studies have indicated that executive function is related to academic performance (Ma et al., 2025), work outcomes (Balconi et al., 2020; Tan et al., 2022), and daily functioning (Suchy et al., 2023), and that impairments in executive functions are commonly observed in several neuropsychological and psychiatric conditions, such as attention-deficit/hyperactivity disorder (ADHD; Chan and Langberg, 2024), depression (He et al., 2025), anxiety (Shields et al., 2016), and addictive behaviors (Kräplin et al., 2022; Zhou and Bai, 2023). Therefore, it is important to explore effective approaches to enhancing executive function.

Among various intervention strategies, physical exercise has attracted increasing attention due to its low cost, high accessibility, and broad cognitive benefits (Kekäläinen et al., 2023; Singh et al., 2025). Compared with long-term exercise programs, acute exercise interventions offer a time-efficient and practical approach and have been associated with immediate changes in executive function (Heath and Shukla, 2020; Samani and Heath, 2018).

Acute exercise intervention is a single bout of physical exercise designed to examine its immediate effects on cognitive or behavioral outcomes (Heath and Shukla, 2020). Previous studies have evaluated the effects of an acute exercise interventions with varying durations, intensities, and types on executive function, with evidence indicating small to moderate positive effects. Despite early meta-analytic evidence indicating that the cognitive benefits of exercise were relatively limited and lacked strong supporting evidence (Cox et al., 2016), recent research has provided converging support for these effects (Ishihara et al., 2021; Tsuk et al., 2019). A study has shown that an acute moderate-intensity resistance exercise significantly improves both attention and executive function in healthy young adults, whereas an acute moderate-intensity aerobic exercise (MIAE) primarily enhances executive function (Tsuk et al., 2019). Another meta-analysis indicated that an acute high-intensity exercise (≥77% HRmax) results in a small immediate improvement in executive function (*d* = 0.24), with an effect size comparable to that observed following longer-duration moderate-intensity exercise. However, this improvement is significant only relative to seated rest (*d* = 0.34). Furthermore, high-intensity interval training (HIIT) produces small acute gains in executive function (*d* = 0.24) at intensities of 77–88% HRmax, with intervention durations of 5–15 min, and these benefits can be observed within 30 min post-exercise (Moreau and Chou, 2019). Although the cognitive benefits of high-intensity exercise have been supported by accumulating evidence (Liu et al., 2024), findings across studies remain heterogeneous. Wang et al. (2013) reported that high-intensity exercise may even impair executive function; specifically, performance on the Wisconsin Card Sorting Test (WCST) declined when participants exercised at approximately 80% of heart rate reserve (HRR). In contrast, other studies have reported that both HIIT and MIAE can enhance cognitive performance, as evidenced by significantly reduced reaction times in the Flanker and Stroop tasks (Dupuy et al., 2018; Yue et al., 2025). These discrepancies may be attributable to variations in exercise intensity regulation and exercise modalities. These differences in exercise intensity and modality may lead to variations in physiological and perceptual responses—such as blood lactate levels, heart rate, respiratory exchange ratio, and perceived exertion—which could contribute to the differential effects of HIIT and MIAE on executive function (Dupuy et al., 2018; Leavitt and DeLuca, 2010; Yue et al., 2025). In light of the inconsistent findings in previous studies, the effects of acute exercise on executive function remain to be further clarified. Accordingly, the present study aimed to systematically investigate the effects of HIIT and MIAE on executive function.

Previous research on the effects of brief acute exercise on executive function still has several limitations. For example, early studies have predominantly employed cognitive tasks such as the Flanker task, Go/No-Go task, Wisconsin Card Sorting Test (WCST), Stroop task, N-back task, and Antisaccade task to assess executive function (Çakaloğlu et al., 2025; Zhou and Bai, 2023). However, it has been argued that these tasks are likely to involve multiple non-executive components in addition to executive processes, giving rise to the well-known issue of task impurity. For instance, the Stroop task relies heavily on language processing and visual perception, and these non-executive components may, to some extent, confound the interpretation of the true effects of an acute exercise on executive function (Zhou and Bai, 2023). Furthermore, prior studies examining the effects of an acute exercise interventions on executive function have tended to focus on a single executive subcomponent (e.g., inhibitory control, working memory, or cognitive flexibility), with relatively few studies systematically investigating the combined effects of an acute exercise across multiple executive function subcomponents (Dai et al., 2024). Therefore, it is necessary to examine and compare the effects of HIIT and MIAE on multiple executive function subcomponents.

In summary, further research is needed to examine the differential effects of acute HIIT and MIAE on executive function. Given the multidimensional nature of executive function, behavioral paradigms that selectively index distinct executive subcomponents offer an effective approach for capturing acute exercise–induced cognitive changes. Accordingly, the present study employed a battery of cognitive tasks to assess core components of executive function, including inhibitory control, working memory, and cognitive flexibility (Çakaloğlu et al., 2025). Specifically, inhibitory control was assessed using the Flanker task, which requires participants to identify the direction of a central target while inhibiting interference from adjacent incongruent flankers. Working memory was evaluated using the 2-back task, which involves continuous updating and monitoring of information held in working memory. Cognitive flexibility was measured using the More–Odd Switching task, in which participants alternate between numerical judgment rules, thereby indexing task-switching ability and associated switching cost. Undergraduates were assigned to one of three groups: a HIIT group, a MIAE group, or a non-exercise Control group. Participants in the exercise groups completed a single 20 min bout of exercise, whereas those in the Control group remained sedentary. Executive function performance was assessed before and after the intervention using the Flanker, 2-back, and More–Odd Switching tasks. Based on prior findings on an acute exercise and executive function, we hypothesized that both HIIT and MIAE would lead to improvements in executive function, as reflected by reduced Flanker interference effects, faster reaction times in the 2-back task, and decreased task-switching cost in the More–Odd Switching task.

Additionally, several potential factors may underlie the differential effects of HIIT and MIAE on executive function. First, HIIT has been shown to induce greater increases in blood lactate levels, which may serve as an alternative energy substrate for neurons and may facilitate synaptic plasticity and prefrontal cortex activation (Jacob et al., 2022). Second, MIAE appears to promote more stable increases in cerebral blood flow, which may support sustained cognitive processing and executive control (Liu et al., 2023). Third, the higher physiological load associated with HIIT may induce transient fatigue or metabolic stress, which has been proposed as a factor contributing to variability in cognitive outcomes (Lubans et al., 2016; Malik et al., 2018; Stute et al., 2020). Taken together, these differences suggest that HIIT and MIAE may be associated with distinct patterns of effects on executive function. Accordingly, the present study aimed to examine and compare their potential differential effects. In contrast, participants in the Control group were expected to show no significant changes in executive function performance from pre-test to post-test. Therefore, significant interaction effects among intervention condition (HIIT vs. MIAE vs. Control), time (pre-test vs. post-test), and executive function task were anticipated.

## Methods

### Ethics statement

This research was approved by the Ethical Committee of Hezhou University. All participants provided written informed consent prior to participation and participated in the experiment voluntarily. The present study was performed in full compliance with the Declaration of Helsinki.

### Participants

**Figure 1:**
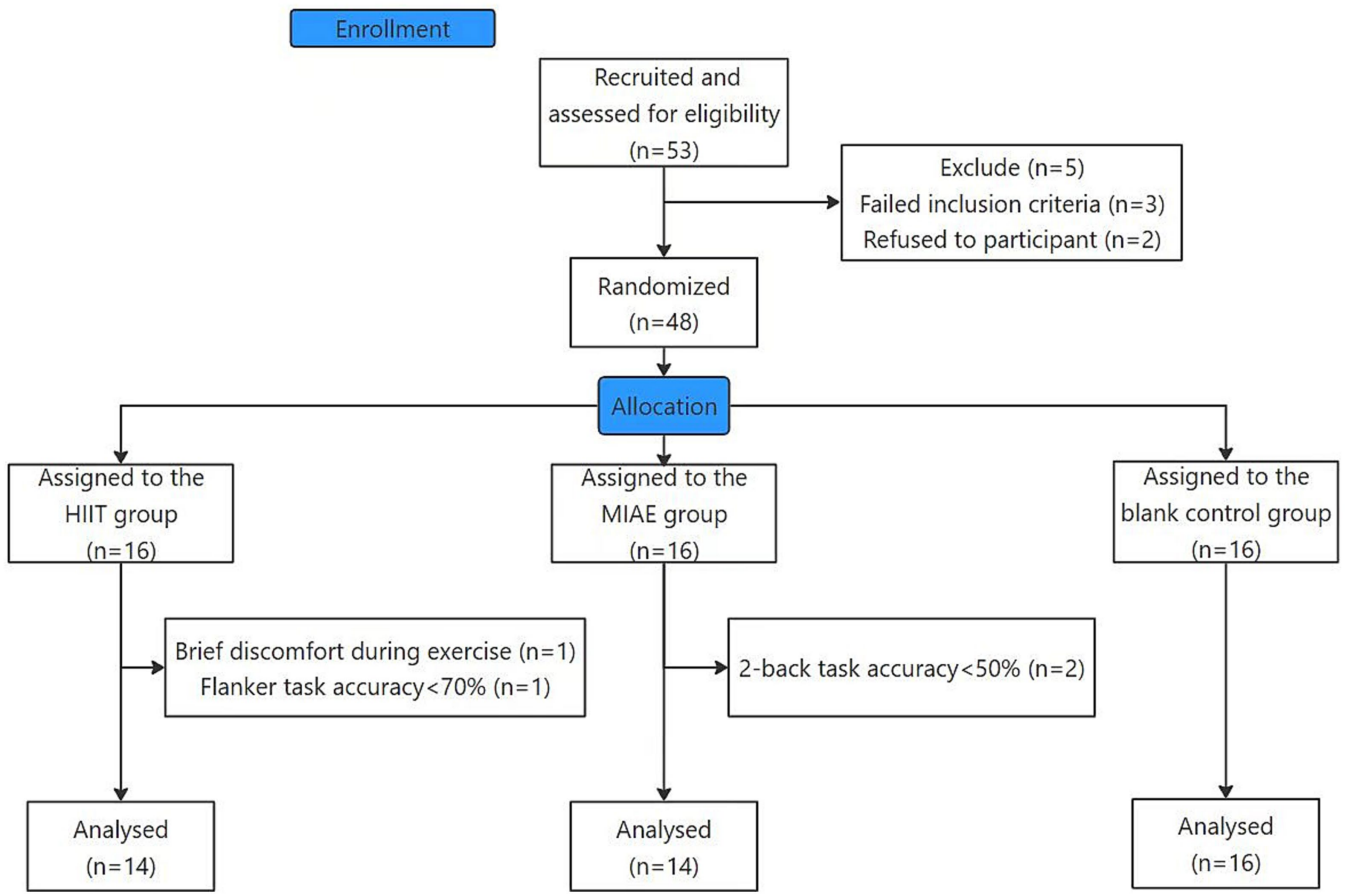
Article inclusion flow chart.

*A priori* power analysis was conducted using G*Power (3.1.9.7) for a 3 × 2 mixed-design ANOVA. Assuming a medium effect size (*f* = 0.25), an alpha level of 0.05, and a statistical power of 0.80, the required total sample size was 42 participants (14 per group). Considering the potential positive effects of exercise on cognition, we recruited only students from non-sports-related majors to minimize the influence of prior exercise experience on the experimental outcomes. A total of 48 participants were recruited (Figure 1). Although the *a priori* power analysis indicated the need for a larger sample size, the present study was conducted as an exploratory investigation. Participants were randomly assigned to one of three groups (16 per group) using a randomization list generated in Excel. Specifically, a random number was generated for each participant using the = RAND() function. We copied these values and pasted them as static values to fix the random numbers, then matched the participant IDs with these fixed random numbers, and finally sorted the list accordingly. The first 16 participants in the shuffled list were allocated to the HIIT group, the next 16 to the MIAE group, and the remaining 16 to the Control group. They were aged 18–21 years and had no prior experience in related experiments. For exclusion screening purposes, participants’ anxiety and depression levels were assessed using the Self-Rating Anxiety Scale (SAS; Yin et al., 2017; Zung, 1971) and the Self-Rating Depression Scale (SDS; Yin et al., 2017; Zung, 1965). Participants with a standard score of 50 or above on SAS or 53 or above on SDS were excluded from further analysis. All participants had normal or corrected-to-normal vision, with no reported color blindness. In addition, none of them had exercise-related discomfort, cardiovascular disease, traumatic brain injury, neurological or psychiatric disorders, or been taking medication. Demographic and related information are presented in Table 1.

**Table 1:** Participant demographics and characteristics.

Group	Age (years)	Sex ratio (M/F)	BMI (kg/m^2^)	SAS score	SDS score	HR (bpm)
HIIT (*N* = 14)	19 (1.5)	9:5	22 (2.0)	32 (5.7)	35 (6.8)	173 (5.5)
MIAE (*N* = 14)	19 (1.1)	1:1	20 (3.4)	27 (4.3)	32 (6.3)	140 (6.1)
Control (*N* = 16)	19 (1.0)	3:1	21 (2.5)	31 (7.7)	35 (8.1)	–

### Apparatus and tools

The Flanker task, the 2-back task, and the More–Odd Switching task were used to assess the three subcomponents of executive function: inhibitory control, working memory, and cognitive flexibility, respectively. All tasks were programmed using E-Prime 2.0 (PST, United States). The hardware tool employed for the behavioral task was a Lenovo laptop running on Windows 11. The screen resolution was 1920 × 1,080 with a refresh rate of 60 Hz. Stimuli were displayed on a gray background (RGB: 153, 153, 153). The Flanker paradigm was used in which participants responded via keypress to the direction (left or right) of the central arrow in a five-arrow array. Stimuli consisted of congruent trials, in which the flanking arrows pointed in the same direction as the target (e.g., <<<<<, >>>>>), and incongruent trials, in which the flanking arrows pointed in the opposite direction (e.g., <<> < <, > > <>>). No neutral condition was included. Each trial began with a 500-ms fixation cross, followed by stimulus presentation for 2,000 ms. Congruent and incongruent trials were presented in a randomized and balanced order. Participants were instructed to respond as quickly and accurately as possible. Reaction time and accuracy were recorded. The task comprised 60 trials, including 12 practice trials. The 2-back task was used to assess participants’ working memory performance for maintaining and updating visual stimulus sequences. Letters were presented sequentially on the screen, and participants were required to compare the currently presented letter with the one presented two trials earlier. Participants pressed the “F” key if the two letters were identical and the “J” key if they were different. For example, in the sequence A–B–A–C, the third letter (A) matched the first and required an “F” response, whereas the fourth letter (C) differed from the second (B) and required a “J” response. Stimuli were presented at a fixed pace, with each letter displayed for 2,500 ms and an interstimulus interval of 500 ms. The More–Odd Switching task was used to assess cognitive flexibility by requiring participants to apply different decision rules based on stimulus color (Liu and Zhang, 2025). In the first block, following a fixation cross, green digits (RGB: 0, 255, 0) were presented, and participants judged parity (odd: “F”; even: “J”). In the second block, following fixation, red digits (RGB: 255, 0, 0) were presented, and participants judged numerical magnitude (less than 5: “F”; greater than 5: “J”). In the third block, the two tasks were combined, with green and red digits presented intermixed, requiring participants to flexibly switch between rules and respond according to the corresponding stimulus color. Each trial began with a 500-ms fixation cross, followed by the presentation of a digit stimulus for 2,500 ms. The task comprised a total of 164 trials, including 36 practice trials. Participants’ health status was assessed using the Chinese version of the Physical Activity Readiness Questionnaire (PAR-Q).

### Procedure

The experimental design of the present study is a two-factor mixed design with Group (HIIT, MIAE, and Control) as between-subject factor and Time (pre-test vs. post-test) as a within-subject factor. We monitored participants’ HR using a heart rate sensor (Polar H10, Finland). When participants’ heart rates did not reach the target range, adjustments were made to the exercise protocol: in the high-intensity interval group, exercise frequency was modified, whereas in the moderate-intensity exercise group, resistance was adjusted using the Keep app connected to the cycle ergometer. During the warm-up period, heart rate in both the HIIT and MIAE groups did not exceed 50% of each participant’s HRmax (220-age). For the HIIT group, participants completed a standardized warm-up protocol lasting 5 min, consisting of jumping jacks (30 s per set, 2 sets), high-knee pulls (8 repetitions per set, 2 sets), walking lunges (8 repetitions per set, 4 sets), arm circles (8 repetitions per set, 4 sets), and wrist and ankle rotations (8 repetitions per set, 4 sets). After the warm-up, participants performed high-intensity interval exercise (80–90% HRmax) for 20 min. Participants performed the HIIT protocol, which comprised burpees, squat jumps, mountain climbers, and high-knee running in a fixed sequence. The training consisted of four circuits, with 15 s of passive recovery between exercises. Upon completion of one circuit, participants were allowed 2 min of recovery before beginning the next circuit. For the MIAE group, participants performed moderate-intensity aerobic exercise on a bicycle ergometer (Keep K0103A, China). The exercise session consisted of a 5 min warm-up period, followed by 20 min of moderate-intensity exercise at 60–70% of HRmax, and a 5 min cool-down period. During moderate-intensity exercise, participants were asked to limit the revolution speed between 55 and 65 r/m. The resistance of the bicycle ergometer would be adjusted to ensure participants reach and maintain 60–70% of their maximum heart rate. At the end of 20 min of moderate-intensity exercise, participants were given a 5 min cool-down period. For the Control group, participants were instructed to remain seated quietly for 20 min and to refrain from using mobile phones or engaging in any activities that might interfere with executive function (Wilmer et al., 2017). The order of the three cognitive tasks was counterbalanced across participants using a Latin square design, and the task order was kept identical for each participant at pre- and post-test. Participants were tested in a quiet room with constant lighting. After reading the experimental instructions and receiving a brief introduction to the equipment, participants adjusted the chair height for comfort and were instructed to respond as quickly and accurately as possible. The assessment tasks lasted approximately 25 min. The study was conducted over a period of 2 months, and all testing sessions were scheduled between 10:00–12:00 and 14:00–18:00.

### Statistical analysis

A repeated-measures ANOVA was used to examine the main effects of group and time, as well as their interaction. When the interaction effect was significant, simple effects analyses were conducted. The independent-samples t-test was used to compare heart rate during exercise between the HIIT and MIAE groups. For the HIIT group, only the average heart rate during the high-intensity intervals was included in the analysis. The Shapiro–Wilk test was used to assess the normality of the data. For variables that did not meet the assumption of normality, the Wilcoxon signed-rank test was applied. Paired-samples *t*-tests were conducted to examine within-group changes and to explore potential trends in performance across the different groups. Statistical analyses were performed using Jamovi (version 2.7.15). Uniform data-screening procedures were applied to all cognitive tasks. Only correct-response trials were included in reaction time analyses. Trials with reaction times below 200 ms or exceeding 1,500 ms were excluded. Additionally, to avoid potential speed–accuracy trade-offs in the Flanker task, accuracy was included in the analysis. Participants with an overall accuracy below 70% in any task were excluded from subsequent analyses. Based on the exclusion criteria, data from four participants were excluded from the analyses. One participant in the HIIT group experienced transient discomfort during training, and another participant’s performance on the flanker task showed an accuracy rate lower than 70%. In the MIAE group, two participants had accuracy below 50% on the 2-back task during the experimental trials. Consequently, data from these participants were excluded from the analysis.

## Results

Normality was assessed using the Shapiro–Wilk test. For reaction time, the MIAE group in the Flanker conflict effect (*W* = 0.78, *p* = 0.003) and the Control group in the 2-back task (*W* = 0.87, *p* = 0.037) showed significant deviations from normality.

For accuracy rate, significant violations of normality were observed across most conditions. Specifically, all groups in the Flanker congruent condition (HIIT: *W* = 0.50, *p* < 0.001; MIAE: *W* = 0.67, *p* < 0.001; Control: *W* = 0.65, *p* < 0.001) demonstrated substantial departures from normality, likely reflecting ceiling effects. Similar deviations were observed in the Flanker incongruent condition (HIIT: *W* = 0.86, *p* = 0.034), the 2-back task (MIAE: *W* = 0.84, *p* = 0.018; Control: *W* = 0.86, *p* = 0.022), and the More-Odd Switching task (MIAE non-switch trials: *W* = 0.79, *p* = 0.005).

For HR, the mean HR in the HIIT group was significantly higher than that in the MIAE group, *t*(26) = 14.90, *p* < 0.001, Cohen’s *d* = 5.62.

For accuracy rate in the congruent Flanker task, the ANOVA revealed no significant main effect of Time, *F*(1, 41) = 0.86, *p* = 0.375, *η^2^ₚ* = 0.02, nor a main effect of Group, *F*(2, 41) = 0.02, *p* = 0.974, *η^2^ₚ* = 0.00. The Time × Group interaction was not significant, *F*(2, 41) = 0.58, *p* = 0.564, *η^2^ₚ* = 0.02.

For reaction time in the congruent Flanker task, the ANOVA revealed significant main effect of Time, *F*(1, 41) = 6.53, *p* = 0.014, *η^2^ₚ* = 0.13, but not Group, *F*(2, 41) = 0.18, *p* = 0.833, *η^2^ₚ* = 0.00. Participants showed shorter reaction times at post-test compared with pre-test, *t*(41) = 2.56, *p* = 0.014. The Time × Group interaction was not significant, *F*(2, 41) = 0.26, *p* = 0.768, *η^2^ₚ* = 0.01.

For accuracy rate in the incongruent Flanker task, the ANOVA revealed no significant main effect of Time, *F*(1, 41) = 2.72, *p* = 0.107, *η^2^ₚ* = 0.06, nor a main effect of Group, *F*(2, 41) = 2.35, *p* = 0.108, *η^2^ₚ* = 0.10. The Time × Group interaction was not significant, *F*(2, 41) = 0.07, *p* = 0.925, *η^2^ₚ* = 0.00.

For reaction time in the incongruent Flanker task, the ANOVA revealed a significant main effect of Time, *F*(1, 41) = 13.85, *p* < 0.001, *η^2^ₚ* = 0.25, but not Group, *F*(2, 41) = 0.16, *p* = 0.846, *η^2^ₚ* = 0.00. Reaction times in the incongruent Flanker task were significantly shorter at post-test than at pre-test, *t*(41) = 3.72, *p* < 0.001. The Time × Group interaction was not significant, *F*(2, 41) = 0.42, *p* = 0.655, *η^2^ₚ* = 0.02.

For the Flanker conflict effect, the ANOVA revealed a significant main effect of Time, *F*(1, 41) = 4.63, *p* = 0.037, *η^2^ₚ* = 0.10, but not Group, *F*(2, 41) = 0.08, *p* = 0.923, *η^2^ₚ* = 0.00. The conflict effect was significantly smaller at post-test than at pre-test, *t*(41) = 2.15, *p* = 0.037. The Time × Group interaction was not significant, *F*(2, 41) = 2.02, *p* = 0.146, *η^2^ₚ* = 0.09.

For accuracy rate in the 2-back task, the ANOVA revealed a significant main effect of Time, *F*(1, 41) = 20.91, *p* < 0.001, *η^2^ₚ* = 0.33, but not Group, *F*(2, 41) = 2.54, *p* = 0.091, *η^2^ₚ* = 0.11. Accuracy rate was significantly higher at post-test than at pre-test, *t*(41) = −4.57, *p* < 0.001. The Time × Group interaction was not significant, *F*(2, 41) = 0.09, *p* = 0.905, *η^2^ₚ* = 0.00.

For reaction time in the 2-back task, the ANOVA revealed a significant main effect of Time, *F*(1, 41) = 9.58, *p* = 0.004, *η^2^ₚ* = 0.18, but not Group, *F*(2, 41) = 1.76, *p* = 0.184, *η^2^ₚ* = 0.07. Reaction times were significantly shorter at post-test than at pre-test, *t*(41) = 3.10, *p* = 0.004. The Time × Group interaction was not significant, *F*(2, 41) = 0.09, *p* = 0.912, *η^2^ₚ* = 0.00.

For accuracy rate in non-switch trials under the More-Odd Switching task, the ANOVA revealed no significant main effect of Time, *F*(1, 41) = 0.45, *p* = 0.505, *η^2^ₚ* = 0.01, nor a main effect of Group, *F*(2, 41) = 0.43, *p* = 0.650, *η^2^ₚ* = 0.02. The Time × Group interaction was not significant, *F*(2, 41) = 0.35, *p* = 0.703, *η^2^ₚ* = 0.01.

For reaction time in non-switch trials under the More-Odd Switching task, the ANOVA revealed a significant main effect of Time, *F*(1, 41) = 13.25, *p* < 0.001, *η^2^ₚ* = 0.24, but not Group, *F*(2, 41) = 0.23, *p* = 0.791, *η^2^ₚ* = 0.00. Reaction times in non-switch trials were significantly shorter at post-test than at pre-test, *t*(41) = 3.64, *p* < 0.001. The Time × Group interaction was not significant, *F*(2, 41) = 2.10, *p* = 0.136, *η^2^ₚ* = 0.09.

For accuracy rate in switch trials under the More-Odd Switching task, the ANOVA revealed a significant main effect of Time, *F*(1, 41) = 11.38, *p* = 0.002, *η^2^ₚ* = 0.21, but not Group, *F*(2, 41) = 0.44, *p* = 0.642, *η^2^ₚ* = 0.02. Accuracy rate in switch trials were significantly higher at post-test than at pre-test, *t*(41) = −3.37, *p* = 0.002. The Time × Group interaction was not significant, *F*(2, 41) = 1.28, *p* = 0.288, *η^2^ₚ* = 0.05.

For reaction time in switch trials under the More-Odd Switching task, the ANOVA revealed a significant main effect of Time, *F*(1, 41) = 44.30, *p* < 0.001, *η^2^ₚ* = 0.52, but not Group, *F*(2, 41) = 0.13, *p* = 0.878, *η^2^ₚ* = 0.00. Reaction times in switch trials were significantly shorter at post-test than at pre-test, *t*(41) = 6.66, *p* < 0.001. The Time × Group interaction was not significant, *F*(2, 41) = 2.20, *p* = 0.124, *η^2^ₚ* = 0.09.

For the switch cost under the More-Odd Switching task, the ANOVA revealed a significant main effect of Time, *F*(1, 41) = 17.33, *p* < 0.001, *η^2^ₚ* = 0.29, but not Group, *F*(2, 41) = 0.04, *p* = 0.956, η^2^ₚ = 0.00. Switch cost was significantly smaller at post-test than at pre-test, *t*(41) = 4.16, *p* < 0.001. The Time × Group interaction was significant, *F*(2, 41) = 5.15, *p* = 0.010, *η^2^ₚ* = 0.20. Follow-up simple effects analyses (with Bonferroni-adjusted *p*-values) showed that switch cost significantly decreased from pre-test to post-test in the HIIT group, *t*(41) = 3.60, *p* = 0.013, and in the MIAE group, *t*(41) = 3.61, *p* = 0.012, whereas no significant change was observed in the Control group (*p* > 0.05) (Tables 2, 3).

**Table 2:** Means and standard deviations of executive function measures across cognitive tasks for all participant groups.

Reaction time (ms)	Group	Pre-test	Post-test	*t*	*p*	Cohen’s *d*
Congruent Trials in Flanker	HIIT	420(55.5)	407(59.7)	1.71	0.112	0.45
	MIAE	415(58.1)	399(56.2)	1.37	0.195	0.36
	Control	415(45.9)	390(40.3)	1.67	0.116	0.41
Incongruent Trials in Flanker	HIIT	503(55.5)	467(55.1)	3.29	0.006	0.88
	MIAE	504(85.9)	465(63.3)	2.01	0.065	0.53
	Control	485(52.9)	464(55.8)	1.56	0.138	0.39
Conflict Effect in Flanker	HIIT	83.5(45.5)	60.7(33.1)	2.04	0.062	0.54
	MIAE	88.6(65.4)	66.2(32.7)	*W* = 81.00	0.014	0.78
	Control	70.3(50.3)	74.3(38.3)	−0.35	0.726	−0.08
2-back	HIIT	997.1(192.4)	924(191.0)	1.81	0.092	0.48
	MIAE	980(211.1)	887(165.9)	1.52	0.152	0.40
	Control	1,085(120.2)	982(185.9)	*W* = 105.00	0.058	0.54
Non-switch Trials in More-Odd Switching	HIIT	591(91.3)	576(70.8)	0.88	0.395	0.23
	MIAE	603(130.7)	581(107.8)	1.61	0.131	0.43
	Control	637(79.7)	568(58.4)	3.96	0.001	0.99
Switch Trials in More-Odd Switching	HIIT	928(62.9)	803(92.1)	5.97	<0.001	1.59
	MIAE	947(162.7)	802(151.9)	4.83	<0.001	1.29
	Control	917(108.7)	853(95.8)	1.74	0.070	0.48
Task-Switching Cost in More-Odd Switching	HIIT	336(52.7)	227(74.2)	5.57	<0.001	1.49
	MIAE	344(127.5)	234(113.6)	3.39	0.005	0.90
	Control	280(89.6)	284(95.7)	−0.14	0.889	−0.03

Due to non-normal distributions in some dependent variables, pre–post comparisons within the HIIT, MIAE, and Control groups were analyzed using the Wilcoxon signed-rank test. Effect sizes for the Wilcoxon tests were reported using the rank-biserial correlation coefficient.

**Table 3:** Means and standard deviations of executive function measures across cognitive tasks for all participant groups.

Accuracy rate	Group	Pre-test	Post-test	*t*	*p*	Cohen’s *d*
Congruent Trials in Flanker	HIIT	0.98(0.024)	0.97(0.058)	*W* = 4.50	0.586	0.50
	MIAE	0.97(0.030)	0.98(0.066)	*W* = 6.00	0.388	−0.42
	Control	0.99(0.027)	0.96(0.078)	*W* = 10.00	0.588	−0.33
Incongruent Trials in Flanker	HIIT	0.91(0.080)	0.94(0.085)	*W* = 14.00	0.333	−0.37
	MIAE	0.92(0.082)	0.94(0.084)	−0.77	0.453	−0.20
	Control	0.87(0.088)	0.89(0.081)	−2.01	0.065	−0.24
2-back	HIIT	0.82(0.120)	0.88(0.084)	−2.11	0.054	−0.56
	MIAE	0.76(0.063)	0.82(0.060)	*W* = 13.00	0.014	−0.75
	Control	0.79(0.114)	0.87(0.081)	*W* = 9.50	0.008	−0.81
Non-switch Trials in More-odd Switching	HIIT	0.96(0.048)	0.96(0.97)	0.24	0.811	0.06
	MIAE	0.94(0.048)	0.95(0.050)	*W* = 7.00	0.268	−0.50
	Control	0.94(0.062)	0.94(0.059)	−0.25	0.803	−0.06
Switch Trials in More-odd Switching	HIIT	0.90(0.087)	0.93(0.063)	−1.52	0.152	−0.40
	MIAE	0.86(0.112)	0.93(0.070)	−2.37	0.034	−0.63
	Control	0.88(0.074)	0.92(0.045)	−1.87	0.080	0.46

Due to non-normal distributions in some dependent variables, pre–post comparisons within the HIIT, MIAE, and Control groups were analyzed using the Wilcoxon signed-rank test. Effect sizes for the Wilcoxon tests were reported using the rank-biserial correlation coefficient.

## Discussion

The present study observed changes in cognitive performance following both HIIT and MIAE. Specifically, both exercise groups showed reductions in switch cost on the More-Odd switching task, whereas no clear changes were observed in working memory performance. In the Flanker task, the exercise groups demonstrated decreases in reaction times under the incongruent condition; however, these changes should be interpreted cautiously. More specifically, the HIIT group showed a reduction in switch cost and a tendency toward shorter reaction times during switch trials. In addition, descriptive statistics indicated a reduction in reaction times in the incongruent Flanker condition and in the switching task in the HIIT group, with a similar pattern observed in the MIAE group. However, given the absence of significant Group × Time interaction effects, these observed changes should be interpreted cautiously and are more likely attributable to practice effects rather than intervention-related improvements in executive function. However, no significant between-group differences were found in executive function measures, indicating that the observed changes were not specific to either intervention condition and do not provide sufficient evidence to support differential effects between HIIT and MIAE.

Descriptive statistics indicated reductions in reaction time in the incongruent condition in the HIIT group, which is consistent with previous findings (Hatch et al., 2021). A similar pattern was observed for the Flanker conflict effect, in line with prior study (Liu et al., 2024). The MIAE group also showed a comparable pattern of changes in the Flanker conflict effect, consistent with earlier report on acute moderate-intensity aerobic exercise (Davranche et al., 2009). However, given the absence of significant Group × Time interaction effects, these findings should be interpreted cautiously and cannot be taken as evidence of intervention effects of exercise on inhibitory control. Recent studies have also reported similar findings. For example, Jiménez-Roldán et al. (2025) found no significant Group × Time interaction effects following a 12-week HIIT intervention using the Stroop task. Taken together, these findings suggest that effects of high-intensity interval exercise on executive function are not consistently observed across studies and may depend on factors such as intervention characteristics, measurement tasks, and lifestyle-related variables. Previous studies have suggested that HIIT protocols may be associated with elevated physiological stress responses, including fatigue and lactate accumulation, which have been considered as potential factors when interpreting inhibitory control performance (Lubans et al., 2016; Malik et al., 2018; Stute et al., 2020). According to Malik et al. (2018), excessive metabolic stress has been proposed as a potential factor that transiently impairs executive function, particularly inhibitory processes requiring high levels of cognitive control. In the present study, a 20-min high-intensity interval protocol was employed at 80–90% of heart rate reserve, with passive recovery during intervals. In contrast, the protocol used by Malik et al. (2018) alternated high-intensity exercise with moderate-intensity exercise. These design differences may be associated with variations in physiological load and fatigue, which have been considered as potential factors in interpreting variability in cognitive performance across the present study.

For working memory, a significant main effect of Time was observed for both reaction time and accuracy rate. Analysis of pre–post reaction times within each group revealed no significant changes in the HIIT and MIAE groups, whereas the Control group showed a marginally significant decrease. Furthermore, a significant increase in accuracy rate was observed across all three groups, potentially due to practice effect. These results suggest that a single 20-min session of HIIT or MIAE may have limited effects on working memory, consistent with a single previous study (Yamazaki et al., 2018). Yamazaki et al. (2018) reported no significant improvement in working memory performance after a 10-min exercise session, and no significant effect of task difficulty. Nevertheless, the magnitude of working memory improvement appeared to be influenced by individual differences, with greater gains observed among individuals with lower baseline performance. Unfortunately, our findings are inconsistent with some previous studies reporting beneficial effects of acute exercise on working memory performance (Çakaloğlu et al., 2025; Singh et al., 2025). The inconsistency between these findings and the present results may be attributable to differences in experimental procedures and the timing of cognitive assessments. Specifically, in the present study, heart rate measurements following a 5 min seated rest, were administered prior to the 2-back working memory task. In contrast, one study reported that the positive effects of acute exercise on working memory primarily occur within 30 min after exercise rather than immediately after exercise cessation (Kamijo and Abe, 2019). Moreover, previous findings suggest that administering multiple cognitive assessments after exercise may increase overall cognitive load, thereby attenuating the beneficial effects of acute exercise on working memory. This may account for the significant reduction in reaction time observed in the Control group at post-test. Similarly, in the present study, the inclusion of additional physiological and cognitive measurements prior to the working memory task may have increased participants’ cognitive burden, potentially offsetting the positive effects of acute exercise. In addition, Liu et al. (2024) found that acute HIIT has minimal effects on working memory, while interventions over 8 weeks produce significant improvements. In the study by Jiménez-Roldán et al. (2025), a 12-week HIIT intervention on the Digit Span Test (DST) showed only a marginal Group × Time interaction (*p* = 0.081). This finding may reflect variability in effect estimates across studies and highlights the potential influence of factors such as measurement tasks, individual differences, and lifestyle-related variables reported in the literature. Furthermore, participants’ fitness levels may also influence working memory improvements, providing an additional explanation for the performance changes observed in the Control group. Exercise may consume cognitive resources that are also involved in performing multiple cognitive tasks, thereby affecting performance on the working memory task. Mou et al. (2023) demonstrated that improvements in working memory are closely associated with individual fitness level, such that participants with lower fitness levels showed significant enhancements following 20 min of moderate-intensity aerobic exercise. In the present study, however, no significant differences in baseline working memory performance were observed across groups, and participants generally reported regular exercise habits, suggesting a relatively adequate level of physical fitness. This may have limited the magnitude of acute exercise–induced improvements in working memory.

For cognitive flexibility, significant improvements were observed in both the HIIT and MIAE groups, as evidenced by reduced reaction time and switch cost in the More-Odd Switching task. This finding is consistent with previous research demonstrating that both acute HIIT and MIAE can effectively enhance cognitive flexibility. Existing evidence further suggests that cognitive benefits are particularly stable in special populations, such as older adults and individuals with lower fitness levels (Chang et al., 2015; Tsai et al., 2016), which may be explained by the compensation model (Voelcker-Rehage and Niemann, 2013). In addition, studies involving young adults have similarly reported improvements in cognitive flexibility following acute exercise, suggesting that these effects are not limited to specific populations. According to the arousal hypothesis, acute exercise increases cerebral blood flow and elevates arousal and executive functioning, thereby facilitating cognitive task performance (Yamazaki et al., 2018). Moreover, acute exercise has been shown to increase brain-derived neurotrophic factor (BDNF) levels and modulate neurotransmitter systems, including dopamine, norepinephrine, and serotonin, which may further support improvements in cognitive performance from a neurophysiological perspective (Kujach et al., 2020). Additionally, HIIT has been shown to induce greater increases in blood lactate levels (Jacob et al., 2022), whereas MIAE appears to promote more stable increases in cerebral blood flow (Liu et al., 2023). These physiological differences may partially explain the superior performance observed in cognitive flexibility. Taken together, the observed enhancements in cognitive flexibility following both HIIT and MIAE interventions in the present study may be attributed to increased post-exercise arousal and blood lactate levels and enhanced cerebral blood flow. In addition, we found that reaction times in non-switch trials significantly decreased in the control group, which may suggest an interaction between practice effects and intervention effects. One possible explanation is that practice effects may have greater potential in the Control group. Compared with the exercise groups, participants in the control group may have experienced lower cognitive load when performing relatively simple tasks, leading to faster responses in non-switch trials. However, as task demands increased in switch trials, this practice-related benefit appeared to diminish, possibly due to the higher cognitive load associated with cognitive flexibility. Another possible explanation is that the control condition, consisting of 20 min of seated rest without engagement, may have introduced factors such as boredom or reduced attentional engagement, which should be considered when interpreting subsequent cognitive outcomes.

Our study also has important limitations. First, we adopted a conservative reaction time limit. Due to an excessive number of null response trials in the pilot study and considering the interaction of task loads, we extended the original response window for each task by 1,000 ms. This adjustment may have reduced the sensitivity of the tasks and potentially confounded the interpretation of our results. This may indicate that the cognitive tasks imposed a certain cognitive load, potentially affecting participants’ reaction times. In addition, manual tasks may be influenced by various confounding factors (Zhou and Bai, 2023), which could compromise the accuracy of the findings. Therefore, future studies may consider using eye-tracking tasks to provide a more precise assessment of cognitive benefits following an acute exercise. Third, all participants in the present study were undergraduate students majoring in non-sports-related fields, whose educational background, lifestyle, and daily physical activity patterns may differ from other populations. This may impose potential limitations on the external validity of the findings. Future research is needed to examine whether the present results can be generalized to more diverse populations. Fourth, future studies investigating the effects of acute exercise on cognitive function should carefully control for variables such as physical fitness, physical activity levels, and psychological state, as these potential confounding factors may influence the effects of acute exercise on executive function. Fifth, practice effects may have contributed to improvements from pre- to post-test, potentially exaggerating the cognitive benefits of acute exercise. In addition, only heart rate was measured as a physiological indicator, and the lack of additional physiological data represents another limitation of the present study. We were unable to comprehensively assess all subcomponents of executive function. For example, inhibitory control comprises distinct dimensions, including conflict inhibition and response inhibition, which are typically measured using different tasks. Response inhibition is commonly assessed using tasks such as the stop-signal task and the anti-saccade task, whereas conflict inhibition is typically measured using tasks such as the Stroop task and the Flanker task. Future research should employ a broader range of cognitive tasks to more thoroughly examine the differential cognitive benefits of acute exercise. Additionally, we acknowledge that the control condition of ‘sitting still for 20 min without devices’ might lead to boredom or mental fatigue, which could influence subsequent cognitive outcomes. To obtain a clearer picture of the exercise intervention’s efficacy, future research should consider using more engaging control activities, such as watching videos or doing light stretches. Finally, although the study met the minimum required sample size, four participants were excluded *post hoc*, which may have reduced the statistical power to detect interaction effects. Given that the interaction effect was the primary outcome of interest, this reduction in sample size may have increased the risk of Type II error. This should be considered as a limitation of the present study.

The present study provides insights into the relationship between acute exercise and executive function. First, the current results are broadly consistent with previous findings suggesting that acute exercise may be associated with changes in executive function, including cognitive flexibility and inhibitory control (Çakaloğlu et al., 2025; Chang et al., 2015; Tsai et al., 2016). However, given the absence of a significant Group × Time interaction effect for inhibitory control, these findings should be interpreted with caution. Second, whereas many previous studies have primarily focused on the effects of acute exercise on a single component of executive function, the present study extends prior research by examining the effects of acute exercise at different intensities on multiple executive function subcomponents. Third, although changes were observed in certain subcomponents, particularly cognitive flexibility, these effects were not specific to the exercise conditions. Therefore, the overall impact of acute exercise on executive function in the present study appears to be selective and limited.

## Conclusion

In summary, the present study provides preliminary evidence that acute exercise may be associated with changes in certain components of executive function, particularly cognitive flexibility. A positive trend was also observed in inhibitory control following the exercise intervention; however, these findings should be interpreted with caution. Given that no significant Group × Time interactions were found for inhibitory control and working memory, the observed changes cannot be definitively attributed to the exercise intervention and may reflect non-specific factors such as repeated testing or other uncontrolled influences.

Overall, the effects of acute exercise in the present study appear to be selective and limited. While some favorable trends were observed in the exercise conditions, similar patterns across groups suggest that these changes may not be specific to the intervention. These findings provide preliminary insights into the relationship between acute exercise and executive function in undergraduate students. Future research should incorporate larger samples, more sensitive cognitive measures, and improved control conditions to better clarify these associations.

## Data Availability

The datasets presented in this study can be found in online repositories. The names of the repository/repositories and accession number(s) can be found in the article/Supplementary material.
