# Supplementary material for: The effects of an acute exercise on executive function in Chinese undergraduate students
Source: Front Psychol. 2026 May 19;17:1853294. doi: 10.3389/fpsyg.2026.1853294 (PMC13226120; doi:10.3389/fpsyg.2026.1853294)
Supplement: Supplementary file 2 [file Supplementary_file_1.DOCX]

**Exercise risk screening questionnaire**

Hello! This questionnaire on Health and Physical Activity aims to understand your health status, lifestyle habits, and physical activity behaviors. Please complete the questionnaire truthfully based on your own situation. The collected data will be used for academic research purposes only, and all personal information will be kept strictly confidential. Thank you very much for your time and cooperation!

Please draw "√" under the serial number of your chosen answer or fill in "___".

1. Name _________

2. Gender: (1) Male (2) Female

3. Ethnicity

(1) Han (2) Other ___

4. Date of birth ___

5. How is your sleep?

(1) Poor (2) Average (3) Good

6. Your usual daily sleep (including naps) is: ___ hours, ___ minutes

7. Do you smoke?

(1) Yes (2) Quitted (3) No, if answer "no", skip to question 9.

8. Your smoking situation: (fill in only 1 item)

(1) Have quit smoking; Stopped smoking for ___ years, and smoked for ___ years before

(2) Smoking now, but not every day; Smoking for about ___ years, now the average smoking volume is ___ cigarettes/week

(3) Smoking every day now; Smoking for about ___ years, now the average smoking volume is ___ cigarettes/day

9. Do you drink alcohol?

(1) Yes (2) No, if answer "no", skip to question 11

10. How much alcohol have you drunk on average each time over the past two weeks? (Can be filled in)

(1) ___ bottles of beer, ___ ml per bottle

(2) Red wine or wine ____ taels

(3) Spirits (liquor, foreign wine, etc.) ___ taels

11. How did you feel in the month before filling out the form?

(1) Very good (2) Good (3) Average (4) Poor (5) Very poor

12. Did you feel stressed in the month before filling out the form?

(1) More than 15 days (2) 7-15 days (3) 1-6 days (4) None (5) Not clear

13. Do your immediate family members have cardiovascular diseases?

(1) None

(2) Yes, the age of onset of the disease in the immediate family is under 60 years old

(3) Yes, the age of onset of the disease in the immediate family is over 60 years old

14. Have you self-tested or been told by medical staff that you have high blood pressure?

(1) No (2) Yes

15. Have you ever been told by your medical staff that you have dyslipidemia (hyperlipidemia)?

(1) No (2) Yes

16. Current or previous medical conditions that you have been told by a doctor to have (multiple options are available)

(1) Heart disease (2) Peripheral vascular disease (3) Cerebrovascular disease

(4) Hypotension (5) Rheumatic fever

(6) Liver disease (excluding fatty liver) (7) Type I or II diabetes mellitus

(8) Chronic obstructive emphysema

(9) Interstitial lung disease or cystic fibrosis (10) Abnormal thyroid function(11)Kidney disease (12)Asthma (13)No of the above diseases

17. Do you have any of the following signs or symptoms (multiple options are available)

(1) palpitations or tachycardia (2) orthopnea or nocturnal paroxysmal dyspnea (3) obvious heart murmur

(4) Abnormal fatigue or shortness of breath during quiet or mild strenuous activities (5) Ankle injury and swelling

(6) Frequent chest discomfort (7) Dizziness or fainting (especially during exercise)

(8) No of the above signs or symptoms

18. Have you engaged in regular exercise and fitness activities in the past 1 year?

(1) No, if answering "no", skip to question 22 (2) Yes

1. Fill in your fitness status according to the different intensity of exercise and whether you are engaged in strength exercises (multiple selections are possible)

| 19-  1 | Is it a high-intensity endurance workout? (Shortness of breath, significantly faster heartbeat, and more sweating during exercise)  (1) Yes. If you select this option, continue to answer the questions in the right column  (2) No | Tempering items | Times / month | Time (minutes) | Years of adherence (years) |
| --- | --- | --- | --- | --- | --- |
|  |  |  |  |  |  |
|  |  |  |  |  |  |
|  |  |  |  |  |  |
| 19-  2 | Is it a moderate-intensity endurance workout? (Rapid breathing, heartbeat, and slight sweating during exercise)  (1) Yes. If you select this option, continue to answer the questions in the right column  (2) No | Tempering items | Times / month | Time (minutes) | Years of adherence (years) |
|  |  |  |  |  |  |
|  |  |  |  |  |  |
|  |  |  |  |  |  |
| 19-  3 | Have you engaged in low-intensity endurance workouts? (Breathing and heartbeat during training are not much different from those when not exercising)  (1) Yes. If you select this option, continue to answer the questions in the right column  (2) No | Tempering items | Times / month | Time (minutes) | Years of adherence (years) |
|  |  |  |  |  |  |
|  |  |  |  |  |  |
|  |  |  |  |  |  |
| 19-  4 | Have you ever engaged in strength training? (Exercise the muscles of the upper limbs, lower limbs, chest and back, neck and other parts)  (1) Yes. If you select this option, continue to answer the questions in the right column  (2) No | Tempering items | Times / month | Time (minutes) | Years of adherence (years) |
|  |  |  |  |  |  |
|  |  |  |  |  |  |
|  |  |  |  |  |  |

20.Do you notice changes in heart rate during exercise?

(1) Yes (2) No

21. Have you ever thought about asking a professional to give you scientific guidance on your sports and fitness?

(1) Yes (2)No

22. What is the reason you don't participate in sports and fitness? (If you have already participated, do not answer this question.) This question can be multiple)

(1) Dislike (2) No time (3) No venue equipment (4) Unsuitable physical condition

(5) Insufficient financial ability (6) Lack of technical guidance (7) Lack of sports companions (8) Fear of being laughed at (9) Others

23. Investigation of static activity behavior

Your average daily TV viewing time is: ___ hours___ minutes

Your average daily use of your computer is: ___ hours, ___ minutes

The average amount of time you spend reading (paper books) per day is: ___ hours___ minutes

The average amount of time you spend playing video games per day (excluding computer games): ___ hours___ minutes

Other sitting-based activities (excluding sleeping and eating): ___ hours___ minutes

1. (This question is limited to women) Whether you have amenorrhea ?

(1) No

(2) Yes, the age at onset of amenorrhea was ___ years.

1. Have you been told by your doctor that you have the following diseases? If so, fill in information such as time of illness, family history, and laboratory results.

|  | Not  Suffer from it  Sick  (1) Yes  (2) No | Long illness  (year) | What are your treatments? (multiple selections are available).  (1) Take medicine  (2) Dietary control  (3) Exercise  (4) Surgery  (5) Others | Do your parents have the disease?  (1) Yes, it occurs under the age of 60  (2) Yes, it occurs over 60 years old  (3) No | Recent laboratory and examination results |
| --- | --- | --- | --- | --- | --- |
| Diabetes |  |  |  |  | Fasting blood glucose: mmol/L |
| High blood pressure |  |  |  |  | Systolic blood pressure: mmHg  Diastolic blood pressure: mmHg |
| Dyslipidemia |  |  |  |  | Triglycerides: mmol/L  Total cholesterol: mmol/L  LDL : mmol/L  HDL : mmol/L |
| Fatty liver |  |  |  |  | (1) Mild (2) Moderate (3) Severe |
| Osteoporosis |  |  |  |  |  |
| Coronary heart disease |  |  |  |  |  |
| (Brain) stroke |  |  |  |  |  |
| Osteoarthritis |  |  |  |  |  |
| Anemia |  |  |  |  | Hemoglobin: g/L |
|  |  |  |  |  |  |

**Exercise survey**

Please recall whether you have engaged in regular physical exercise in stages. Fill in the exercise items, practice frequency and time in the table below. If not, fill in "None" in the Workout section. If you have already filled in the specific information in question 19, please skip this question.

| Time  Activity |  |  |  |  |  |  |  |  |  |
| --- | --- | --- | --- | --- | --- | --- | --- | --- | --- |
|  | Exercise duration ( months) | Frequency  ( times per month) | Duration per session ( minutes) | Exercise duration ( months) | Frequency  ( times per month) | Duration per session ( minutes) | Exercise duration ( months) | Frequency  ( times per month) | Duration per session ( minutes) |
|  |  |  |  |  |  |  |  |  |  |
|  |  |  |  |  |  |  |  |  |  |
|  |  |  |  |  |  |  |  |  |  |
|  |  |  |  |  |  |  |  |  |  |
| Notes: |  |  |  |  |  |  |  |  |  |

Physical activity hazard checklist

Table 1 Physical activity hazard checklist

|  | Inspect the item | Answer | |
| --- | --- | --- | --- |
| 1 | Has the doctor said that there is a heart problem (is the ECG abnormal)? | yes | no |
| 2 | Can't breathe and have chest pain during exercise? | yes | no |
| 3 | Do you have chest pain and irregular heartbeat when you don't exercise? | yes | no |
| 4 | Do you have dizziness or loss of consciousness? | yes | no |
| 5 | Is there a family history of sudden death of unknown causes? | yes | no |
| 6 | Has the doctor ever said that there is a foot and back disorder? | yes | no |
| 7 | Can lower back pain get worse during exercise? | yes | no |

Note: This table is used for self-assessment of physical condition before each exercise

Even if the subject answers "yes" in one of the items, the risk of exercise will increase, and a doctor should be consulted before exercise. If all answers are "no", you need to take the following pre-exercise self-examination questionnaire and adopt an appropriate exercise plan.

Table 3 Pre-exercise self-examination form

|  | Inspect the item | Answer | |
| --- | --- | --- | --- |
| 1 | There is a strong pain in the feet and lower back | yes | no |
| 2 | Fever | yes | no |
| 3 | Weakness and weakness in the body | yes | no |
| 4 | Nausea, want to vomit | yes | no |
| 5 | Headache, dizziness | yes | no |
| 6 | Tinnitus | yes | no |
| 7 | Physical fatigue | yes | no |
| 8 | Lack of sleep, poor health | yes | no |
| 9 | No appetite | yes | no |
| 10 | Drunk for two days in a row and unwell | yes | no |
| 11 | Abdominal pain caused by diarrhea or constipation | yes | no |
| 12 | A little exercise makes you out of breath | yes | no |
| 13 | Cough, phlegm, seems to be cold | yes | no |
| 14 | Chest pain | yes | no |
| 15 | There are signs of heat stroke | yes | no |

Note: This table is used for self-assessment of physical condition before each exercise. If the answer is "yes" before the exercise, you need to terminate today's activity. If the answer is "no" before exercise, the activity is carried out within a reasonable range.
